# Chemical Analysis and Molecular Modelling of Cyclodextrin-Formulated Propofol and Its Sodium Salt to Improve Drug Solubility, Stability and Pharmacokinetics (Cytogenotoxicity)

**DOI:** 10.3390/ph16050667

**Published:** 2023-04-28

**Authors:** Benedikt Wilhelms, Jens Broscheit, Sergey Shityakov

**Affiliations:** 1 Department of Anaesthesiology, Intensive Care, Emergency and Pain Medicine, Würzburg University Hospital, 97080 Würzburg, Germany; 2 Infochemistry Scientific Center, Laboratory of Chemoinformatics, ITMO University, Saint Petersburg 191002, Russia

**Keywords:** propofol, anaesthesiology, HPβCD, ^1^H-NMR spectroscopy, calorimetry, molecular modelling, cytotoxicity, genotoxicity

## Abstract

Propofol is a widely used general anesthetic in clinical practice, but its use is limited by its water-insoluble nature and associated pharmacokinetic and pharmacodynamic limitations. Therefore, researchers have been searching for alternative formulations to lipid emulsion to address the remaining side effects. In this study, novel formulations for propofol and its sodium salt Na-propofolat were designed and tested using the amphiphilic cyclodextrin (CD) derivative hydroxypropyl-β-cyclodextrin (HPβCD). The study found that spectroscopic and calorimetric measurements suggested complex formation between propofol/Na-propofolate and HPβCD, which was confirmed by the absence of an evaporation peak and different glass transition temperatures. Moreover, the formulated compounds showed no cytotoxicity and genotoxicity compared to the reference. The molecular modeling simulations based on molecular docking predicted a higher affinity for propofol/HPβCD than for Na-propofolate/HPβCD, as the former complex was more stable. This finding was further confirmed by high-performance liquid chromatography. In conclusion, the CD-based formulations of propofol and its sodium salt may be a promising option and a plausible alternative to conventional lipid emulsions.

## 1. Introduction

Propofol (2,6-diisopropyl phenol) is mainly used for the induction and maintenance of anesthesia during surgery and endoscopy, as well as for short-term sedation in intensive care. Its rapid onset of action, low accumulation when applied intravenously, and rare side effects have made it one of the most commonly used narcotics worldwide, and it was included in the WHO’s list of essential medicines in 2016 [1]. The drug primarily has a hypnotic effect and shows dose-dependent sedative, anxiolytic, and amnestic effects, and belongs to the group of alkylphenols [2,3]. Due to its lipophilic properties, the drug is marketed in one percent or two percent emulsion form of soybean oil and egg lecithin [4].

Relatively few side effects occur with the drug if it is used correctly in clinical practice. The initially higher risk of an anaphylactic reaction could be reduced by replacing the previously used Kolliphor et al. with soybean oil, but it persists. Despite this change in emulsion, injection pain remains a common side effect and varies in the frequency of occurrence from 28% to 90% [5]. In addition, blood pressure drops, bradycardia, and hyperlipidemia may occur [6]. Increased growth of bacteria and an increased risk of postoperative sepsis in patients were proven for lipid emulsions [7]. Propofol infusion syndrome (PRIS) has been described as a rare but particularly serious side effect. PRIS is defined as a complex of symptoms following propofol infusion, including cardiovascular adverse effects, metabolic acidosis, rhabdomyolysis, and lipemia. Although an association with dosage is assumed, PRIS is also described with propofol application below 4 mg/h/kg of body weight [8]. Furthermore, PRIS can occur without the typical signs of acidosis, rhabdomyolysis, hyperkalemia, or renal failure, making early diagnosis difficult [8].

Due to the frequent use of propofol and the listed side effects, increasing efforts have been made to investigate alternative options to the one percent and two percent propofol lipid emulsion [9]. By varying the lipid content, changes in side effects can be registered. It was shown that by using 0.5% propofol, a significant reduction of injection pain in children could be achieved [10]. In addition to other lipid-based emulsions, non-lipid formulations were also examined. Approaches range from nanoparticle carriers, and prodrugs, to cyclodextrins [9].

The central challenge for non-lipid formulations for intravenous administration is the limited water solubility of propofol (0.150 mg/l), as the benzene ring and the isopropyl groups result in high lipophilicity of propofol (logP = 4.16) [11]. In contrast to other narcotics, which can form aqueous solutions without further problems, the hydroxyl group of propofol with a pKa value of 11 does not form salts in solutions [12]. For a solvent of propofol, these carrier substances themselves mustn’t have any anesthetic effects or toxic properties of their own [12]. Since many of the typical solvents such as propylene glycol and benzyl alcohols have toxic properties, an aqueous solution would be a potentially preferable alternative [13].

Cyclodextrins offer the advantage of binding lipophilic substances in aqueous solutions by means of inclusion complexes [13]. Cyclodextrins are cyclic oligosaccharides composed of α-(1,4)-glycosidically linked α-D-glucopyranose monomers. A complex of the cyclodextrin and the guest molecule is formed by hydrogen bonds and dipole-dipole interactions (Van der Waals forces) [12]. Therefore, the increased solubility facilitates the use of cyclodextrins as carriers at biological barriers without damage to the lipid layers [12,14].

In addition to applications at biological barriers such as the skin or colon, increasingly more studies are being conducted on cyclodextrin-drug complexes at the blood-brain barrier (BBB) [12,14]. Pure β-cyclodextrin is not suitable for intravenous use due to the nephrotoxicity and hemolytic effect demonstrated in mice [15]. For the derivatives of β-CD, a change in the physicochemical properties due to the respective modification was shown [14]. For the two cyclodextrin derivatives HPβCD (hydroxypropyl-beta-cyclodextrin) and sulfobutylether-β-cyclodextrin (SBEβCD), an intravenous application was successfully demonstrated [15]. Both cyclodextrins showed increased water solubility and an almost 15-fold reduction in toxicity compared to β-cyclodextrin [16]. Thus, HPβCD has only reversible renal toxic effects even at high doses due to the increased solubility of the cyclodextrin derivative [14]. However, it has been shown that complex formation of HPβCD with the host is reduced compared to pure β-cyclodextrin [17]. An association between increased substitution of the hydroxypropyl group and reduced complex formation was demonstrated [18,19,20,21].

The host-guest complexes between different cyclodextrin derivatives and propofol have already been described [22,23,24]. The complexes formed with propofol do not require special chemical formulations and are available as clear solutions without the addition of oil. In addition to propofol/HPβCD, the use of propofolate made it possible to establish a venous injectable, stable, easily storable, and pharmacologically active formulation of propofol. The anion of propofol (phenolate) thus incorporated into the complex was patented, including the preparation, in 2011 [25]. This is made possible by the conversion of propofol in an alkaline environment (pH 9–10) to the sodium salt. The molar ratio of propofolate and HPβCD in the complex is specified between 1:2 and 1:4 [25].

In comparison to propofol, propofolate (logP = 0.65) is pharmacologically active and theoretically more hydrophilic. However, the further structural investigation is still needed. The purpose of this work is to analyze the complex formation between propofol and propofolate with HPβCD in terms of stability (Table S1). Through 1H-NMR spectroscopy and molecular modeling, a closer determination of the coordination of the narcotic and the ring of propofol/HPβCD and Na-propofolate/HPβCD can be made. Additionally, LC-MS/MS can be used to determine the pharmacokinetic properties. Thermodynamic parameters are investigated through differential scanning calorimetry (DSC). Cytotoxicity is a decisive criterion for the potential further use of substances in medicine. Therefore, in the present experiments, cytotoxicity tests MTT, LDH assay, and EZ4U were carried out on CerebEND cells previously used to analyze processes at the BBB, as cytotoxic effects for propofol as a lipid emulsion have already been reported on BBB cell lines [26]. Propofol has also been shown to disrupt BBB permeability in the mouse model. The studies by Shityakov et al. demonstrated the permeability at the BBB, which is decisive for narcotics, propofol, and modified β-cyclodextrin complexes [23]. In addition to cytotoxicity, the comet assay can detect double-strand and single-strand breaks as DNA damage and represents a standardized procedure for determining genotoxicity [27]. The cell line HL-60, which has already been established for the comet assay, is used [28]. This work aims to compare the previously non-investigated formulation of Na-propofolate/HPβCD with propofol/HPβCD.

## 2. Results and Discussion

### 2.1. H-NMR Spectroscopy

**Figure 1:**
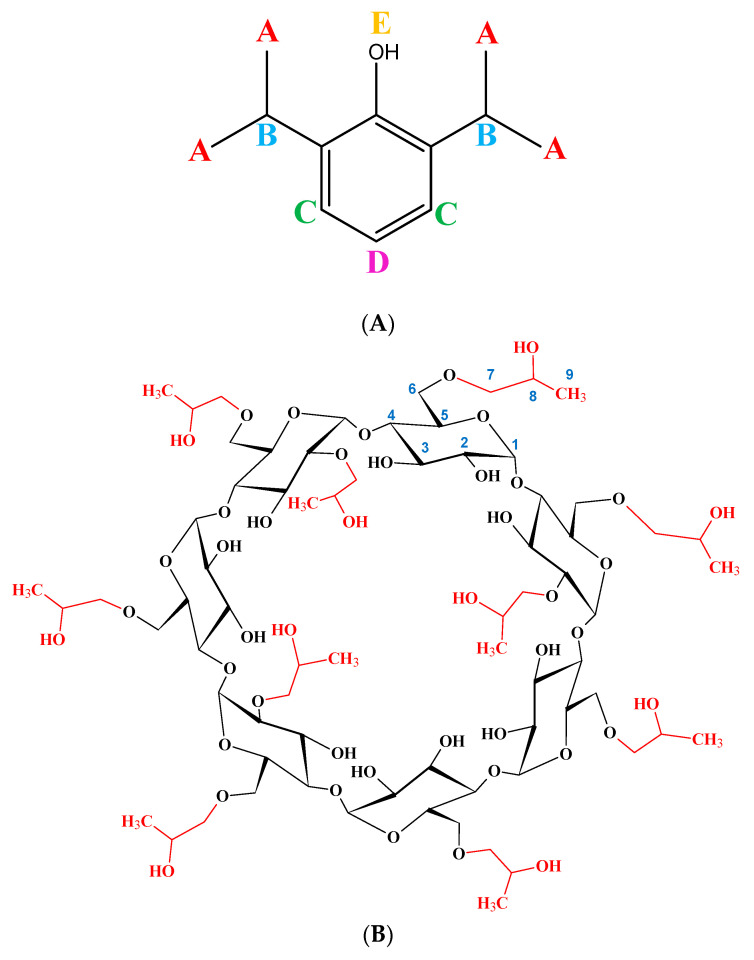
(**A**) Propofol; (**B**) HPβCD. For better orientation, the molecules of propofol and HPβCD are shown separately.

**Figure 2:**
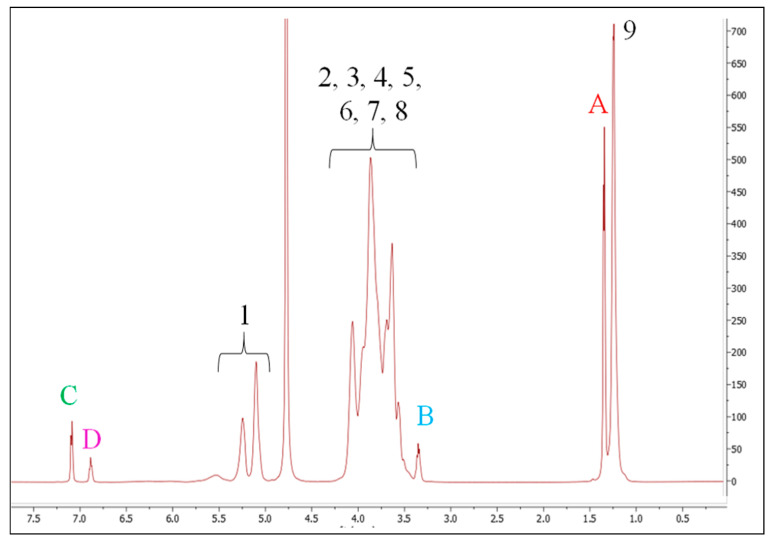
^1^H NMR spectrum of the Na-Propofolat/HPβCD sample at pH 7 (D_2_O, 25 °C, 500 MHz) with peak assignment. (1–9 refers to the signals of HPβCD, **A**–**D** refers to the signals of propofol).

The ^1^H-NMR spectrum of Na-propofolate/HPβCD showed the characteristic peaks and proved the presence of propofol (Figure 1A) and HPβCD (Figure 1B). The ratio of the integral values of the respective signals corresponded to the integral values of both propofol and HPβCD (Figure 2). Due to overlapping signals, a change of chemical shifts between the H-3 and the H-5 of the HPβCD was unable to be determined in this ^1^H-NMR spectrum (Table 1). For complexation of a β-cyclodextrin derivative with a drug, there should be a chemical shift at these positions [29]. However, for the propofol signals of Na-propofolate/HPβCD, a chemical shift resulted in comparison with the ^1^H-NMR spectrum of propofol (Table 1). The increase in the values of the chemical shift for propofol due to complex formation was in line with the experiments conducted by Trapani et al. for propofol/HPβCD and therefore suggests complex formation [24]. The absence of the signal from the hydroxyl group was explained as propofolate was present as an ionised form of propofol in the complex. This corresponds to the patent for propofolate filed in 2012 [25]. The molar ratio of propofol/HPβCD to free HPβCD of 1:2 was determined by Loftsson on the basis of his calculations for water-soluble propofol and solutions of the cyclodextrin derivative [30]. This suggests that, assuming a guest–host ratio of propofol and cyclodextrin of 1:1, in this case, only every third HPβCD is bound in a complex with propofol.

### 2.2. Molecular Modelling

**Figure 3:**
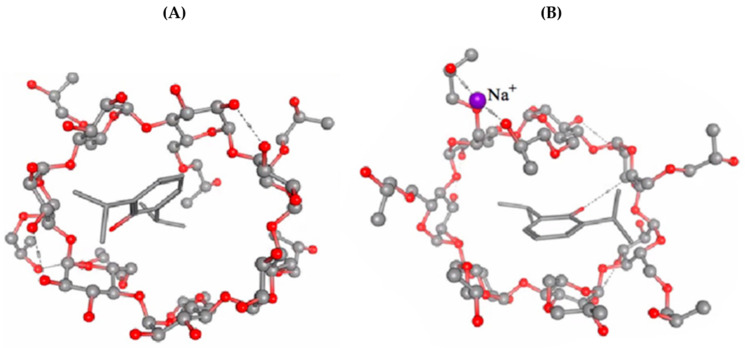
Molecular modelling for (**A**) propofol/HPβCD and (**B**) Na-propofolate/HPβCD using AutoDock v.4.2.5.1, Gibbs free energy value (ΔGbind) in kcal/mol.

The molecular modelling is shown in Figure 3. For complex A, the binding affinity value of −49.54 kcal/mol was lower than for complex B with a value of −34.94 kcal/mol. Thus, an overall increased stability can be predicted for complex A of the propofol/HPβCD compared to complex B of the Na-propofolate/HPβCD. Assuming equal concentrations of propofol/HPβCD and Na-propofolate/HPβCD, it can be expected that in the case of the Na-propofolate, the concentration of free propofol will be higher because, due to the weaker binding of the propofol, the chemical equilibrium is shifted towards the dissociated molecules. Due to the lower binding affinity value of the binding of propofol to the cyclodextrin ring, an overall significantly increased stability of the binding strength was predicted for propofol/HPβCD compared to Na-propofolate/HPβCD. Assuming equal concentrations of propofol/HPβCD and Na-propofolate/HPβCD, it can be expected that in the case of Na-propofolate, the concentration of free propofol was higher, as the chemical equilibrium was shifted towards the dissociated molecules due to the weaker binding of propofol. If the aim was to achieve a longer-acting dose with an equal amount of propofol, the use of the propofol/HPβCD is recommended, as here the binding of the propofol to the complex was stronger. Thus, propofol would be released more slowly and would act over a longer period.

### 2.3. DSC

**Figure 4:**
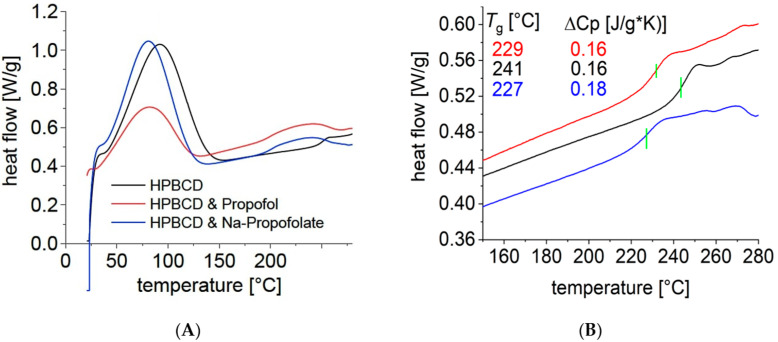
DSC measurements for HPβCD (black), propofol/HPβCD (red), Na-propofolate/HPβCD (blue); DSC 204 F1 Phoenix, volume flow: 20 mL/min N2; heating rate: 10 °C/min (0 °C to 280 °C); first heating (**A**) and second heating (**B**) (*x*-axis: temperature (°C), *y*-axis: specific heat flow (W/g)).

**Figure 5:**
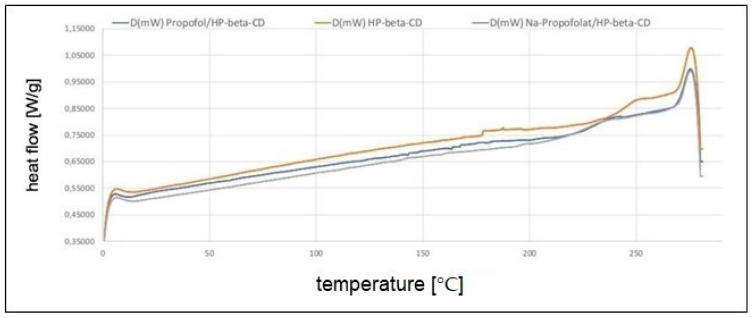
DSC measurements for HPβCD, propofol/HPβCD, and Na-propofolate/HPβCD for the second heating; DSC 204 F1 Phoenix, volume flow: 20 mL/min N2; heating rate: 10 °C/min (0 °C to 280 °C) (x-axis: temperature (°C), y-axis: specific heat flow (W/g)).

Figure 4 and Figure 5 show the results of DSC. In Figure 4A, for the temperature range between 270 and 280 °C, the peak was the same for all three substances. This peak was clearly higher than the boiling point of propofol, which is stated in the literature at 256 °C [20]. There was no difference in this temperature range between the measurements for HPβCD and the propofol-containing substances. Since there was no evidence of evaporation of propofol, it can be assumed that propofol is still bound to the cyclodextrin derivatives. Thus, a complex binding of propofol/HPβCD and Na-propofolate/HPβCD can be assumed.

On the one hand, there was a reduction in the glass transition temperature for the substances with propofol compared to HPβCD, as shown in Figure 4B. It resulted in a higher value of 241 °C for HPβCD than for propofol/HPβCD and Na-propofolate/HPβCD, for which the *Tg* values of 229 °C and 227 °C were close to each other. On the other hand, the DSC measurement suggests that propofol continued to be bound in the complexes propofol/HPβCD and Na-propofolate/HPβCD, even at higher temperatures above the boiling point of 256°, since no peak was shown as an indication for the evaporation of propofol.

The comparatively higher glass transition temperature of HPβCD, compared to other derivatives of β-cyclodextrin, is caused by the substitution of the hydrogen at the hydroxy-propyl group. A chain extension of the molecule results in increased mobility, and thus a lower glass transition temperature [31,32]. HPβCD shows a high glass transition temperature compared to cyclodextrins and other carbohydrates with a higher molecular weight. For example, HPβCD with a molecular weight of Mw = 1400 g/mol is 30 °C above the glass transition temperature of the polysaccharide dextran 10 with Mw = 10,000 g/mol and 90 °C above the glass transition temperature of the methylated β-cyclodextrin with a similar molecular weight of Mw = 1310 g/mol [33].

The high glass transition temperature, which can be regarded as a benchmark for the physical stability of a solid, forms another argument in favor of HPβCD over other cyclodextrins [33]. In this respect, the incorporation of propofol lowers the glass transition temperature, which is evident in the measurements. Together with the absence of the evaporation peak in the DSC measurement, the lowering of the glass transition temperature underpins the complex formation of the propofol with the cyclodextrin derivatives investigated. The molecular modeling is also consistent with complex formation, which for both substances yields enthalpy values typical for HPβCD complexes [34].

Weiler’s physicochemical investigations showed that HPβCD has the greatest potential for stabilizing drug complexes compared to other amorphous carrier substances, including cyclodextrin derivatives [33]. However, the complex formation could also be demonstrated for other derivatives of β-cyclodextrin [35,36]. In the context of this analysis, however, the high stability of the complexes speaks in favor of the use of HPβCD, which is crucial for good drug storability and a basic prerequisite for broad clinical use [33]. Propofol is weakly bound with the binding energies of the hydrogen bonds calculated in this work, so it can be cleaved off during application. Further investigations should determine the complex formation constants to better quantify the cleavage of propofol from the complex.

### 2.4. Quantitative Determination of Propofol and Its CD Complexes by LC–MS/MS

**Figure 6:**
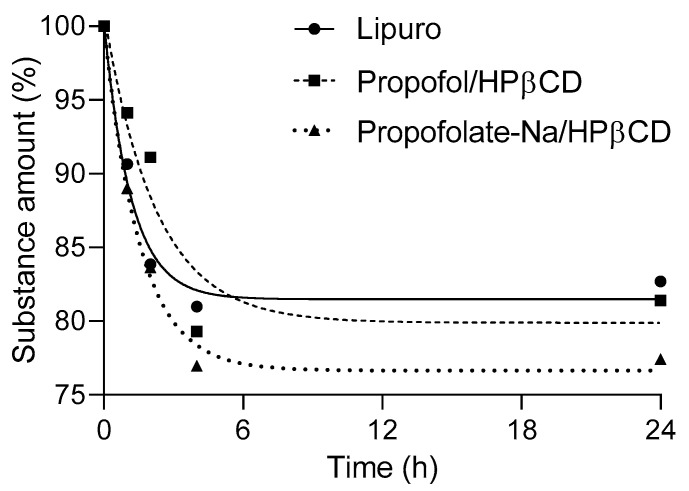
LC–MS/MS results for propofol, propofol/HPβCD, and Na-propofolate/HPβCD at room temperature after 0, 1, 2, 4, and 24 h. Data expressed as mean of detected concentration compared to initial concentration.

Figure 6 and Figure S1 and Table 2 show the results of LC–MS/MS. Using the optimised LC–MS/MS parameters, the propofol amount in all three substances showed comparable results as an indicator of good stability since the concentrations of propofol/HPβCD and Na-propofolate/HPβCD corresponded to the sample of propofol as a lipid emulsion. The increase in propofol concentration between 4 h and 24 h in particular was detected in all three samples with Na-propofolate/HPβCD (77.4%), showing a slightly lower stability than propofol/HPβCD (81.4%). These results agree with the results of DSC and molecular modelling.

### 2.5. Cytoxicity Tests

**Figure 7:**
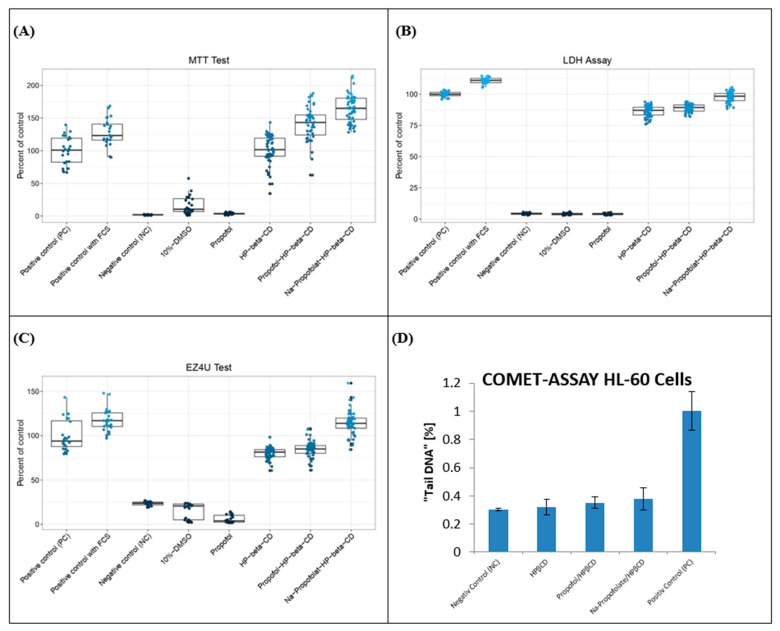
(**A**) MTT test, (**B**) LDH assay, (**C**) EZ4U test (CerebEND cells), and (**D**) the comet assay (HL-60 cells) after 24 h of exposure. MTT Test: PC: 100%, PC with FCS *: 128.2% (padj < 0.001), NC *: 1.9% (padj < 0.001), 10%-DMSO *: 16.4% (padj < 0.001), propofol *: 3.5% (padj < 0.001), HPβCD: 101.6% (padj = 1.0), propofol/HPβCD *: 140.5% (padj < 0.001), Na-propofolate/HPβCD *: 164.5% (padj < 0.001). LDH Assay: PC: 100%, PC with FCS *: 111% (padj < 0.001), NC *: 4.3% (padj < 0.001), 10%-DMSO *: 4% (padj < 0.001), propofol *: 3.9% (padj < 0.001), HPβCD *: 86.2% (padj < 0.001), propofol/HPβCD *: 88.8% (padj < 0.001), Na-propofolate/HPβCD *: 97.9% (padj < 0.001). EZ4U Test: PC: 100%, PC with FCS *: 117.5% (padj < 0.001), NC *: 23.3% (padj < 0.001), 10%-DMSO *: 15.9% (padj < 0.001), propofol *: 5.9% (padj < 0.001), HPβCD *: 80.0% (padj < 0.001), propofol/HPβCD *: 84.8% (padj < 0.001), Na-propofolate/HPβCD *: 114% (padj < 0.001). Comet assay: PC: 100%, NC *: 30.2% (padj < 0.001), HPβCD *: 32.1% (padj = 0.008), propofol/HPβCD *: 35.2% (padj = 0.008), Na-propofolate/HPβCD *: 37.9% (padj = 0.02) (statistically significant marked with *).

Figure 7 provides an overview of the results of the cytotoxicity tests and the comet assay. In all three cytotoxicity tests, the substances examined showed the same order in their cytotoxic properties. Thus, Na-propofolate/HPβCD had the lowest cytotoxicity values, and HPβCD comparatively the highest. For propofol, the three test methods showed a very high cytotoxicity with hardly any remaining vital cells. This is consistent with the publications that demonstrated high cytotoxicity of propofol in various cell lines [37,38]. According to a recent publication, propofol acts as a classical protonophore, whereby the cytotoxicity could be based on the translocation of protons through double lipid layers [39]. Mitochondrial depolarisation would induce apoptosis through the activation of caspase-9, caspase-3, and DNA fragmentation [39]. The extent to which this mechanism can be confirmed has not yet been conclusively clarified.

### 2.6. Cytotoxicity, Genotoxicity, and BBB Permeation Analysis

In this work, the complexation with the cyclodextrin derivative showed a strongly reduced cytotoxicity for the propofol. The effect of cyclodextrins to cause a reduced cytotoxicity of otherwise cytotoxic drugs by complexation has already been described for other drugs [40]. In contrast, there are publications that provide evidence for an enhancement of the cytotoxic potential by cyclodextrin derivatives [41]. For propofol/HPβCD and Na-propofolate/HPβCD, no cytotoxicity resulted for the examined CerebEND cell line in this work.

#### 2.6.1. Cytotoxicity of Propofol at the Blood-Brain Barrier

To conclude the effects of substances on the processes at the blood-brain barrier (BBB), CerebEND cells were selected for the experiments. Apart from the previously mentioned activation of apoptosis, other factors such as the restriction of neuronal differentiation in the mouse model, and the activation of microglia by propofol contribute to the cytotoxicity of propofol for BBB cells [42,43]. The disruption of BBB permeability induced by propofol has also been demonstrated in the mouse model and could be due to the increased activation of heat shock proteins (HSP) by propofol [44]. In this context, efforts to reduce the cytotoxicity of propofol are increasing. Current studies have shown protective effects for two agents used in the treatment of gout, the uricostatic febuxostat, and the uricosuric benzbromarone, against propofol-induced damage to endothelial brain cells [42,45].

#### 2.6.2. Cytotoxicity of β-Cyclodextrin and HPβCD at the Blood-Brain Barrier

The interaction of cyclodextrin derivatives with the BBB varies depending on the size of the cyclodextrin rings. The highest cytotoxicity to BBB endothelial cells was observed for α-cyclodextrins, and the lowest for γ-cyclodextrins [46]. However, there are also clear differences between individual cyclodextrins. β-Cyclodextrin exhibited high cytotoxicity in intestinal Caco-2 cells, while no cytotoxicity was detected in another study for HPβCD in the same cell line at a dosage of the mean inhibitory concentration of up to 200 mmol/l [47,48]. A review specifically investigating the toxic effects of HPβCD confirmed the limited toxicity of this cyclodextrin derivative [49]. Studies conducted in rats, mice, and dogs showed that the substance was well-tolerated, especially for oral administration, while intravenous administration resulted in histopathological changes in the lungs, liver, and kidneys of animals, which were fully reversible [49]. For humans, good tolerability of HPβCD without renal function restriction was reported [49]. In addition to ongoing studies on complex formation between HPβCD and other drugs, the U.S. Food and Drug Administration’s classification of HPβCD as an extremely safe, pharmaceutically inactive carrier substance indicates that HPβCD will continue to be considered for complex formation with drugs [50]. In contrast to β-cyclodextrin, HPβCD also showed no cytotoxicity on endothelial cells of the BBB [51]. This is consistent with the results of this study, which found no cytotoxicity for HPβCD on the CerebEND cell line in all three experiments. This is also consistent with the use of HPβCD in other applications.

#### 2.6.3. Propofol/HPβCD and Na-Propofolate/HPβCD at the Blood–Brain Barrier

The extent to which the cytotoxicity of propofol at the blood–brain barrier is reduced by complex formation with HPβCD cannot be conclusively answered in this work and must be determined in further investigations. However, the results of this work suggest that no increase in the cytotoxicity of the CerebEND cells results from the administration of propofol/HPβCD and Na-propofolate/HPβCD. In this context, these results correspond to other studies that were able to show a reduction in cytotoxicity through complex formation with cyclodextrins [40]. Starting points for further investigations are the dose-dependent examination of cytotoxicity. Additionally, already published options for predicting the cytotoxicity of cyclodextrin complexes at the blood–brain barrier can be used [52]. Another central aspect for the investigation of cyclodextrin complexes is the transport of the narcotic at the blood–brain barrier. In this context, an increased BBB permeability has already been described for other HPβCD inclusion complexes [53]. In her work, Appelt-Menzel investigated the transport speed of propofol/HPβCD and Na-propofolate/HPβCD on BBB endothelial cells. Compared to propofol, both propofol/HPβCD and Na-propofolate/HPβCD showed an up to threefold increase in transport speed in stem-cell-based BBB models [54]. This result forms a central argument for the continuation of detailed investigations of propofol/HPβCD and Na-propofolate/HPβCD at the blood–brain barrier [54].

### 2.7. Comet Assay

**Figure 8:**
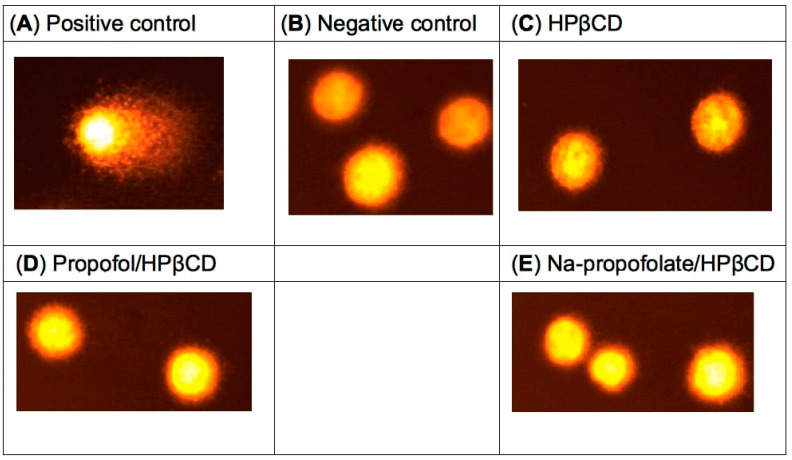
Representative Hl-60 cells during evaluation of the comet assay. (**A**) Positive control, (**B**) Negative control, (**C**) HPβCD, (**D**) propofol/HPβCD, (**E**) Na-propofolate/HPβCD.

The evaluation of the comet assay shows a lack of genotoxic effects for the substances HPβCD, propofol/HPβCD, and Na-propofolate/HPβCD after 24 h exposure at 37 °C on the HL-60 cells (Figure 7 and Figure 8). This corresponds to other studies in which a protective effect associated with β-cyclodextrin derivatives was described in the comet assay [55]. HPβCD also shows no genotoxicity in the DNA synthesis test for measuring DNA damage, in the mouse lymphoma test for detecting gene mutations, and in the human lymphocyte test for detecting a possible chromosomal abnormality [49]. The results of these three tests are in agreement with the results of the comet assay determined in this trial and support the thesis that HPβCD has no genotoxic effect [49].

### 2.8. Alternative Formulations of Propofol

In his 2010 article “Exploring the Frontiers of Propofol Formulation Strategy: Is There Life Beyond the Milky Way?” published in the British Journal of Anaesthesia, Egan questions whether different formulations of propofol can reduce side effects [9]. The extent to which complexes of cyclodextrins and propofol play a major role in this question remains to be seen. The results of the present investigation show that the propofol/HPβCD formulations hold promise for reducing side effects, which can be further explored. On the one hand, this work confirms what Baker et al. described regarding inclusion complexes between cyclodextrins and drugs. The complex formation demonstrated in this work leads to changes in physical, chemical, and biological properties concerning both the drug and the cyclodextrin derivative [12]. For example, in contrast to propofol as a lipid emulsion, there is no longer any cytotoxicity of the propofol bound in the complex with HPβCD.

On the other hand, further work suggests little change in the effect of formulations of cyclodextrin/propofol complexes compared to propofol as a lipid emulsion. Thus, for a formulation of propofol with the cyclodextrin derivative sulfobutyl-ether-β-cyclodextrin (SBEβCD), extensive agreement of the pharmacological and pharmacodynamic properties with propofol as a lipid emulsion was demonstrated in a three-hour infusion in the pig model [56]. Although no directly corresponding study is available for HPβCD, it also seems possible for the propofol/HPβCD complexes to result in an effect comparable to that of propofol. Thus, even matching the already mentioned increased transport speed at the blood-brain barrier, a reduced induction time and a prolonged effect time for propofol/HPβCD was described in comparison to propofol in the mouse model [24,54]. Further studies also show effective transport of propofol with rapid onset of anesthesia for another HPβCD formulation [57]. Based on his work, McIntosh et al. argued for a three-compartment model for the pharmacokinetic description of mammalian propofol/HPβCD, corresponding to Cockshott’s three-compartment model for the analysis of the distribution of propofol as a lipid emulsion [57]. However, the rebinding of the cyclodextrin derivative to other lipophilic substances after desorption of propofol at the target site of action has been described as a challenge for the future use of cyclodextrins [12]. In this context, for example, the muscle relaxant rocuronium is mentioned, for which bindings to cyclodextrins have also been demonstrated [12,58]. This could lead to an undesired shortening of the effect of the muscle relaxant. Detailed studies will thus be necessary to further analyze the pharmacological properties of propofol/HPβCD as well as to investigate potential interactions with other drugs. As a proposal to circumvent these problems, the additional application of pharmaceutically inactive substances that bind strongly to the cyclodextrin derivatives and displace other substances from the complex may be considered.

**Figure 9:**
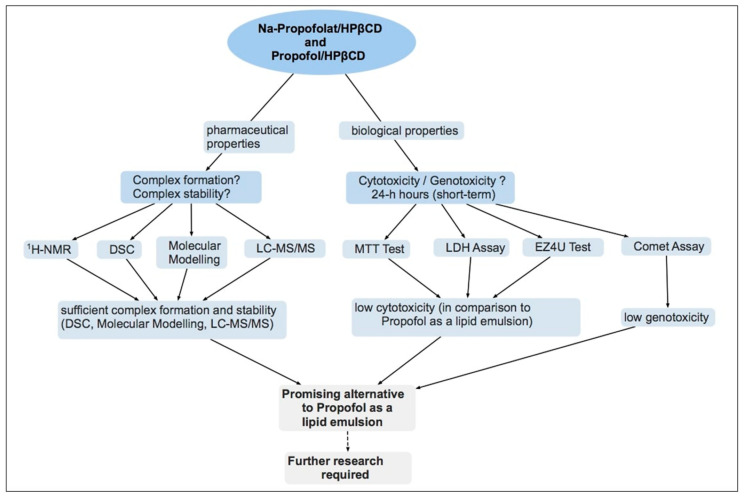
Summary of the approach and the experiments of this study.

Due to the promising situation, initial investigations have already been carried out on the change in the side effects of propofol through cyclodextrin formulations. To date, these have not shown any improvement in the side effect profile. On the contrary, in healthy adults, an increase in injection pain resulted from a propofol compound with sulfobutyl-ether-β-cyclodextrin (SBEβCD) [59]. However, a major advantage of HPβCD is the variety of possible uses, ranging from the described formulation as an aqueous solution suitable for injection to a hypothetical sublingual form of application [30]. The latter is supported not least by the favorable pharmacokinetic conditions described by Loftsson et al. and the good tolerability of oral HPβCD formulations in animal models [30,49]. The reduced induction time, prolonged action time, and increased transport speed at the BBB due to the complexes could lead to a reduction of the dosage, which could condition an improvement of the side effect profile [24,54]. Na-propofolate/HPβCD as a new formulation could provide starting points for further research with HPβCD. When considering the feasibility of the formulations, the decisive criteria will remain the dosage as well as the effect of the drug at the given dosage [30]. In summary, propofol/HPβCD and Na-propofolate/HPβCD, as a new alternative formulation, represent a potentially promising alternative to the use of conventional propofol and should be investigated as an option in further research (Figure 9).

## 3. Material and Methods

### 3.1. ^1^H-NMR Spectroscopy

The measurements were conducted using a Bruker Avance III HD 500 (1H; 500 MHz) spectrometer at 25 °C. Deuterated DMSO-d6 (with a reference signal at 2.5 ppm) was used as a solvent to obtain high-resolution NMR spectra. TMS was used as the reference value. The NMR program MestreNova (Mestrelab Research, A Coruna, Spain) (Version: 12.02-20910) was used to evaluate the spectra for chemical shift and peak integrals.

### 3.2. Molecular Modelling

Molecular docking was used to predict the binding enthalpy values (ΔGbind) of the protein-ligand interactions. The propofol molecule from the PubChem chemical database was used. PyMol v.1.2 software (DeLano Scientific LLC, San Francisco, CA, USA) was used to assess the shape of the cavity of the β-cyclodextrin ring, and the structure for the HPβCD was created using the same software. The software AutoDock v.4.2.5.1 (Scripps Research, San Diego, CA, USA) was used for central modeling, and the software PyMol v.1.2 (DeLano Scientific LLC, San Francisco, CA, USA) was integrated for calculating the binding enthalpy values (ΔGbind) [23,60].

### 3.3. Differential Scanning Calorimetry (DSC)

DSC measurements have been used repeatedly for the analysis of cyclodextrin inclusion complexes [35,36]. HPβCD was assumed to form inclusion complexes with other substances based on DSC [23]. A DSC 204 F1 Phoenix from (NETZSCH, Selb, Germany) with a CC200 F1 controller (NETZSCH, Selb, Germany) was used for the experiments. The samples were heated in an aluminum crucible, and an empty aluminum crucible served as a reference. The measurements were carried out in a nitrogen atmosphere at a volume flow of 20 mL/min and a heating rate of 10 °C/min in the range of 0 °C to 280 °C.

### 3.4. Quantitative Determination of Propofol and Its CD Complexes by LC-MS/MS

The quantitative analyses were performed on a Shimadzu HPLC system (Shimadzu Corporation, Kyoto, Japan) equipped with a binary pump, an autosampler, and a column oven with a switching valve, coupled with a triple-quadrupole mass spectrometer. The LC-MS/MS analysis was controlled by Shimadzu LabSolutions Shimadzu 5.60 SP2 software. Separation was achieved on a Kinetex EVO C18 (100 × 2.1 mm, 5 µm) column, and propofol was eluted using a gradient mobile phase consisting of 10 mM ammonium carbonate buffer pH 9.0 (A) and methanol (B). The column temperature was set to 40 °C, and the injection volume was 5 µL. The flow rate was 0.7 mL/min.

The APCI potential was set to −3 kV, and the ion source temperature was 350 °C. Argon was used as the CID gas with a pressure of 230 kPa. Nitrogen was used as the nebulizing and drying gas; the flow rates were set to 3 L/min and 5 L/min, respectively. The temperature of both the desolvation line and heat block was 200 °C. The instrument was set up in multiple reaction monitoring (MRM) modes; the transition 177.2 → 161.15 was monitored for the quantifier, and the transition 177.2 → 176.6 for the qualifiers.

For the stability study of Propofol emulsion and CD complexes of Propofol at room temperature, complexes were dissolved in PBS to obtain a solution with a concentration of 20 mg/mL. Propofol-Lipuro^®^ was used as a reference. The solutions and emulsion were stored at room temperature for 24 h. Samples were taken after 0, 1, 2, 4, and 24 h; samples were diluted 200 times with a water and methanol mixture in a 1:1 ratio, and the propofol amount was determined by LC-MS/MS.

### 3.5. Cytotoxicity Tests

The CerebEND cells used in this study were provided by Professor Förster from the Department of Anesthesiology at the University of Würzburg. They were obtained as an immortalized, endothelial cell line from the cerebellum of neonatal mice and were morphologically similar to the cerebellum, creating a barrier with cell-to-cell connections with claudin-5, occludin, and VE-cadherin proteins. These cells were particularly suitable for studying processes at the blood-brain barrier. The dose of 26 mg/kg (26 μL/g) for propofol was taken from the literature, and a mouse weight of 25 g was assumed. The lean body mass water content of the mice was estimated to be 78%, based on the work of Widdowson and Dickerson et al. (Table S2).

For all the cytotoxicity tests, the positive control (PC) was cell culture medium, and the measurement results were referenced to the PC. A mixture of 90% cell culture medium and 10% heat-inactivated fetal calf serum (FCS) was added to the cells as an additional positive control. The negative controls were a well series of the microtiter plate without cells (NC) and a solution of 90% cell culture medium and 10% dimethyl sulfoxide (DMSO).

#### 3.5.1. MTT Test

The substances were washed twice with phosphate-buffered saline (PBS) (pH 7.2) and placed in the incubator at 37 °C for 24 h. Then, 100 μL of the light-sensitive MTT working solution, which was prepared by mixing 90 μL of cell culture medium and 10 μL of MTT stock solution (5 mg/mL), was added to each well of the microtiter plate and incubated for four hours. The supernatants were aspirated, and 100 μL of a solution of 14.4 mL isopropanol and 600 μL hydrochloric acid (HCl) (0.1 mol/L) was added to each well of the microtiter plate. After protection from light and circular shaking for ten minutes, the evaluation was photometrically carried out using the Magellan Tecan program at a wavelength of 570 nm.

#### 3.5.2. LDH Assay

For the LDH assay, the substances were placed in an incubator at 37 °C for 24 h. Then, 5 μL of the lysis buffer of the Cytotoxicity Detection Kit of the LDH assay and 100 μL of medium were added to the microtitre plate and placed in an incubator at 37 °C for 15 min. Next, a reaction mix of the catalyst and dye in a ratio of 1:45 was prepared, and 100 μL was pipetted into all wells of the microtitre plate and stored for 10 min under light exclusion at room temperature. Finally, 50 μL of the stock solution of the LDH assay was added and placed on the laboratory shaker for mixing. The measurement was then evaluated at 492 nm using the Magellan Tecan evaluation.

#### 3.5.3. EZ4U Test

After washing twice with phosphate-buffered saline containing Mg2+ and Ca2+ (PBS-Ca-Mg buffer, pH 7.2), the test substances were added to the respective wells of the microtitre plate and placed in an incubator at 37 °C for 24 h. After washing each well of the microtitre plate again with PBS, a solution of 200 μL dimethyl ether (DME) with 20 μL EZ4U reagent was added to each well of the microtitre plate and placed in the incubator for 90 min. Subsequently, the Magellan Tecan programme was used to evaluate the spectrophotometric measurement, which was carried out at a wavelength of 450 nm with a reference wavelength of 590 nm.

### 3.6. Comet Assay

HL-60 cells (human leukemia) were used for the comet assay, provided by Professor Stopper (Department of Pharmacology, University Würzburg). This cell line is increasingly being used to test for cytotoxicity and apoptotic effects and has already been applied for the investigation of propofol [61].

The substances were added to the samples on the microtitre plate. As the negative control, 30 μL H2O, and as the positive control, 50 μmol H_2_O_2_ were chosen. In preparation for electrophoresis, the 6 reaction vessels were each filled with 180 μL of the 0.5% Low Melting Point agarose solution. After incubation for 30 min, 20 μL of HL-60 cells, which were stored in test tubes at 4 °C, were added to the reaction tubes. Again, 45 μL of the cell-agarose mixture was added to the two slides coated with 1.5% High Melting Point agarose solution and covered with coverslips immediately afterwards. After the coverslips were removed after five minutes, the prepared coverslips were able to be stored in the pre-cooled cuvette with the previously prepared lysis solution for at least five minutes. Afterwards, the slides were incubated for 24 h at 4 °C. In the cooled electrophoresis chamber, the slides were covered with the pre-cooled electrophoresis buffer and incubated for 20 min under light exclusion. The electrophoresis was then started at a constant alkaline pH (>13), a voltage of 25 V, and an initial current of 300 mA. Through the electrophoresis, the DNA strand breaks were divided, whereby the negatively charged DNA fragments migrated to the anode. According to the DNA breaks, “tails” of different sizes and brightness formed behind the cells and were able to be measured with the evaluation software.

After electrophoresis, the slides were neutralized three times with neutralization buffer (0.4 M trisaminomethane (Tris) at pH 7.5) to accelerate the rewinding of the DNA. The slides were then fixed with methanol precooled at −20 °C and dried at room temperature for 24 h. After applying a solution to the slides (20 mL) of the fluorescent dye Gel Red and DABCO in a ratio of 1:3, the evaluation was able to be carried out. For each test substance, 100 randomly selected cells (50 cells per two slides) were evaluated with a fluorescence microscope (Labophot 2; Nikon GmbH, Düsseldorf, Germany) at 200× magnification. Results are provided as average ± standard deviation. The tail moment as a decisive parameter was calculated using the evaluation software Komet 5 (BFi OPTiLAS, Gröbenzell, Germany).

### 3.7. Statistical Data on the Cytotoxicity Tests and the Comet Assay

Using the one-sample t-tests, the two-sided hypothesis was used to test whether the arithmetic mean was significantly different from the positive controls (1.0) of the cytotoxicity tests and the comet assay. The distribution was verified graphically. The significance level was set at alpha = 0.05. Where appropriate, p-values were adjusted using the Bonferroni method.

## 4. Conclusions

Despite the widespread clinical use of propofol, efforts to find alternative formulations to the lipid emulsion have increasingly developed due to existing side effects. In this work, the substances propofol/HPβCD and Na-propofolate/HPβCD were investigated as alternative formulations of propofol. The investigation involved several methods including 1H-NMR spectroscopy, molecular modeling, DSC measurements, LC–MS/MS, cytotoxicity tests (MTT test, LDH assay, EZ4U test), and the comet assay as a genotoxicity test. The focus was on biological, chemical, and physical properties and complex formation.

Although the question of complex formation between propofol and HPβCD cannot be answered solely based on 1H-NMR spectroscopy, the DSC measurements suggest a complex formation between propofol and HPβCD due to the different glass transition temperatures. Na-propofolate/HPβCD and propofol/HPβCD show no evaporation peak up to the maximum temperature of 280 °C, despite the lower boiling point of 256 °C for propofol. Molecular modeling suggests that the binding between propofol and cyclodextrin occurs via the isopropyl groups of propofol, with the aromatic ring not penetrating the cavity of the cyclodextrin ring. Enthalpy values obtained from the molecular modeling corresponded in magnitude to weak hydrogen bonds.

For the Na-propofolate/HPβCD, an easier cleavage of propofol from the complex was predicted, whereas, for propofol/HPβCD, the cleavage was expected to be slower but over a longer period. The results of the LC–MS/MS prognosticate promising pharmacokinetic properties in terms of stability since the concentrations of propofol/HPβCD (81.4%) and Na-propofolate/HPβCD (77.4%) corresponded to the sample of propofol as a lipid emulsion (82.7%) after 24 h.

In comparison to the high cytotoxicity of propofol as a lipid emulsion, the cytotoxicity tests performed on the CerebEND as a blood–brain barrier (BBB) cell line showed no evidence of cytotoxicity for HPβCD, propofol/HPβCD, and Na-propofolate/HPβCD after 24 h of exposure. The results show the same order for the three assays with the highest cell viability for the cells treated with Na-propofolate/HPβCD and the lowest cell viability for HPβCD. In the comet assay, there was no genotoxic effect on the HL-60 cell line after 24 h exposure at 37 °C to propofol/HPβCD and Na-propofolate/HPβCD.

The results of this work suggest the continuation of studies of propofol/HPβCD and Na-propofolate/HPβCD as promising options for propofol formulations.

## Figures and Tables

**Table 1:** ^1^H-NMR spectra (DMSO-d6; 500 MHz; 25 °C); chemical shift δ: in ppm.

	Propofol	Na-Propofolate/HPβCD	∆ δ
OH of propofol	s 7.96		
H-2 of propofol (C)	d 6.95	d 7.12	+0.17
H-3 of propofol (D)	dd 6.77	dd 6.91	+0.14
H-4 of propofol (B)	m 3.29	m 3.33	+0.04
H-5 of propofol (A)	d 1.15	d 1.31	+0.16

**Table 2:** LC–MS/MS results for propofol, propofol/HPβCD and Na-propofolate/HPβCD at room temperature after 0, 1, 2, 4, and 24 h. Data expressed as mean of detected concentration compared to initial concentration.

	Time (h)				
	0	1	2	4	24
	Propofol Amount (%)				
Propofol (Lipuro)	100	90.657	83.871	80.991	82.701
Na-propofolat- HPβCD	100	89.011	83.661	76.966	77.433
Propofol-HPβCD	100	94.128	91.122	79.309	81.408

## Data Availability

Data are contained within the article and supplementary materials.

## Supplementary Materials

The following supporting information can be downloaded at: https://www.mdpi.com/article/10.3390/ph16050667/s1, Table S1. Overview of physical and chemical properties of the substances propofol^20^, β-CD^14^ and HPβCD^21^; Table S2. Calculations for the cytotoxicity tests and the comet assay; Figure S1. Gradient program for the elution of propofol; t R = 2.340 min.
