# Supplementary material for: Chemical Analysis and Molecular Modelling of Cyclodextrin-Formulated Propofol and Its Sodium Salt to Improve Drug Solubility, Stability and Pharmacokinetics (Cytogenotoxicity)
Source: Pharmaceuticals (Basel). 2023 Apr 28;16(5):667. doi: 10.3390/ph16050667 (PMC10221165; doi:10.3390/ph16050667)
Supplement: Supplementary file 1 [file pharmaceuticals-16-00667-s001.zip › pharmaceuticals-2201035-supplementary.pdf]

Supplementary Materials

Table S1. Overview of physical and chemical properties of the substances propofol[20], β-CD[14] and HPβCD[21].

**Substances**	**Molecular weight**	**Melting point**	**Boiling point**	***p*Ka Value**
Propofol	178,14 g/mol	13 °C	256 °C	11,1
β-Cyclodextrin	1134,99 g/mol	290 °C	-	-
HPβCD	1541,55 g/mol	278 °C	-	-

Table S2. Calculations for the cytotoxicity tests and the comet assay.

**Propofol dose**	26 mg/kg = 26 µg/g
**mouse weight**	25 g
**Dose of Propofol/Maus**	25g * 26 µg/g = 650 µg/maus
**H_2O-Volume of mice**	25g * 78 % = 19,5 ml
**Propofol concentration**	650 µg/Maus / 19,5 mL = 33,3 µg/mL
**Na-Propofolat/HPβCD concentration**	33,3 µg/mL * 15,15 = 504,5 µg/mL
**Propofol/HPβCD concentration**	33,3 µg/mL * 15,15 = 504,5 µg/mL
**HPβCD concentration**	504,5 µg/mL - 33,3 µg/ml = 471,2 µg/mL

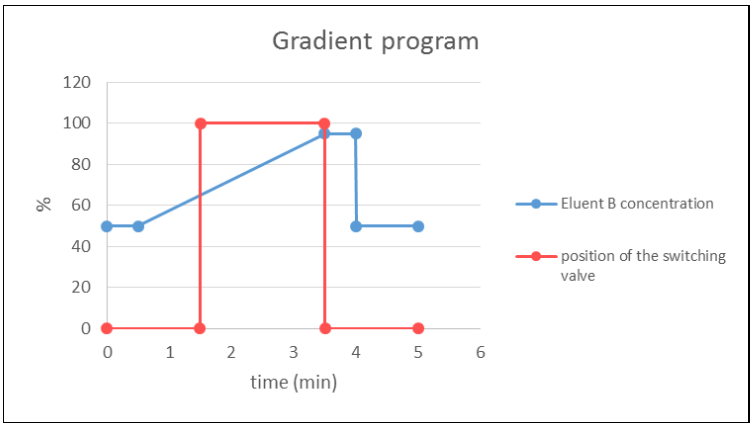

Figure S1. Gradient program for the elution of propofol; t R = 2.340 min.
